# Zinc Finger E-Box Binding Homeobox 2 as a Prognostic Biomarker in Various Cancers and Its Correlation with Infiltrating Immune Cells in Ovarian Cancer

**DOI:** 10.3390/cimb44030079

**Published:** 2022-03-01

**Authors:** Hye-Ran Kim, Choong Won Seo, Sang Jun Han, Jae-Ho Lee, Jongwan Kim

**Affiliations:** 1 Department of Biomedical Laboratory Science, Dong-Eui Institute of Technology, 54 Yangji-ro, Busanjin-gu, Busan 47230, Korea; hrkim@dit.ac.kr (H.-R.K.); seo3711@dit.ac.kr (C.W.S.); 2 Department of Biotechnology, College of Fisheries Sciences, Pukyong National University, 45 Yongso-ro, Nam-gu, Busan 48513, Korea; sjhan@pknu.ac.kr; 3 Department of Anatomy, Keimyung University School of Medicine, 1095 Dalgubeol-daero, Dalseo-gu, Daegu 42601, Korea

**Keywords:** ovarian cancer, ZEB2, prognosis, biomarker, immune cells

## Abstract

This study investigated the expression of zinc finger E-box binding homeobox 2 (ZEB2), its prognostic significance in various cancers, and the correlation between ZEB2 and infiltrating immune cells and ZEB2-related proteins in ovarian cancer (OV). The Gene Expression Profiling Interactive Analysis tool was used to analyze RNA sequencing data and cancer survival rates, based on normal and tumor tissue data available in The Cancer Genome Atlas (TCGA) database. The Kaplan–Meier plotter and PrognoScan databases were used to analyze the prognostic value of ZEB2 in OV (*n* = 1144). The Tumor Immune Estimation Resource was used to investigate the correlation between ZEB2 and infiltrating immune cells in various cancers, including OV. High ZEB2 expression was associated with a poorer prognosis in OV. In OV, ZEB2 is positively correlated with CD8+T cells, neutrophils, macrophages, and dendritic cell invasion; and ZEB2 is negatively correlated with tumor-infiltrating B cells. The STRING database was used to investigate the correlations with ZEB2-related proteins. The results reveal that ZEB2 was positively correlated with SMAD1 and SMAD2 in OV. Our findings may serve as a potential prognostic biomarker, and provide novel insights into the tumor immunology in OV. Thus, ZEB2 may be a potential diagnostic and therapeutic target in OV.

## 1. Introduction

Ovarian cancer (OV) accounts for only 3% of cancers in women. However, it is the fifth most common cause of mortality in women after lung, breast, colorectal, and pancreatic cancers [1]. According to the World Health Organization (WHO), OV is reported as one of the main causes of cancer-related deaths, and is considered a significant source of disease and death in women worldwide [2]. Moreover, OV, a major public health concern, is the most fatal gynecological malignancy, and, despite surgery and aggressive frontline treatments, most cancers ultimately recur, leading to chemotherapy-resistant diseases [3]. Recent studies have shown that this cancer recurs in approximately 50% of patients within 16 months, with a five-year overall survival rate of less than 50% [4]. Although surgery and chemotherapy have advanced significantly, the reported overall survival rates are very low. Therefore, many studies have been conducted to identify the prognostic factors, such as tumor biomarkers, to improve the prognosis of ovarian cancer. Recently, efforts have been made to improve new biomarkers, such as antibodies, cyclic tumor DNA, and micro-RNAs [5].

Zinc finger E-box binding homeobox 2 (ZEB2) gene, which is a member of the ZEB family, encodes the ZFHX1B gene and is located on chromosome 2q22 [6]. ZEB2 is a DNA-binding transcription factor that is mainly involved in epithelial-to-mesenchymal transition (EMT). EMT is a conserved process that transforms a mature epithelial-like state into a mobile mesenchymal state. It is a cellular program that is important for embryonic development, wound healing, and malignant progression of cancer. EMT plays a crucial role in the metastatic dissemination events of cancer cells: EMT confers cancer cells with a motile and invasive phenotype [7,8,9]. In addition to the transforming growth factor-β signaling pathway (TGF-β), which is a key inducer of EMT, the activation of several transcription factors, including ZEB1, ZEB2, SNAI1, SNAI2, TWIST1, and TWIST2, either directly or indirectly, regulate the E-cadherin promoter [10]. ZEB2 is a major transcription factor that promotes EMT under both normal and pathological conditions [11]. Emerging evidence suggests that ZEB2 plays a crucial role in EMT-induced processes, including development, differentiation, and malignant mechanisms, such as drug resistance, cancer stem cell-like characteristics, apoptosis, survival, cell cycle arrest, tumor recurrence, and metastasis [12]. ZEB2 is associated with the progression and development of malignancies such as liver cancer, cervical cancer, gastric cancer, and breast cancer [13,14,15,16,17]. Recent studies have shown that ZEB2 promotes peritoneal metastasis, through the process of regulating invasive cells and tumorigenesis of cancer cells in high-grade serous ovarian cancers (HGSOCs) [18]. In addition, ZEB2 had a significant correlation with poor prognosis and could improve EMT conversion in OV [19]. Thus, we performed a meta-analysis to evaluate the prognostic biomarker value of ZEB2, which is involved in the progression and development of various types of cancer, including OV.

The immune system plays a significant role in the onset and progression of cancer. Recent research has revealed that ZEB2 is expressed by the lymphatic system cells, such as T lymphocytes, B lymphocytes, and NK cells, as well as by the immune cells in myeloid systems, such as dendritic cells and macrophages, and that ZEB2 has the ability to control the transcriptional networks needed for cell differentiation and maintenance [20]. The interaction between ZEB2, which is associated with immune cells, and the immune cells that infiltrate OV is unclear. Thus, we investigated whether there was a correlation between ZEB2 and the infiltrating immune cells in various cancers, including OV.

In the present study, we investigated ZEB2 in various types of cancer, based on The Cancer Genome Atlas (TCGA) data from public databases. We investigated the correlation of ZEB2 with prognosis and infiltrating immune cells in various types of cancer, including OV. Furthermore, we focused the correlation between ZEB2-related proteins in OV. 

## 2. Materials and Methods

### 2.1. Gene Expression Profiling Interactive Analysis (GEPIA)

The GEPIA database, which is a web server tool consisting of normal and tumor tissue samples from the TCGA and GTEx projects [21,22,23], was used to analyze the differences in ZEB2 expression between normal and tumor tissues, based on RNA sequencing.

### 2.2. Kaplan–Meier Plotter Database Analysis

The Kaplan–Meier plotter is based on an online database [24] and is capable of assessing the association of genes with survival in various types of cancer, including ovarian cancer (*n* = 1144). It includes survival rates, such as progression-free survival (PFS), overall survival (OS), and post-progression survival (PPS), and clinical data (including stage, grade, and TP53 mutation). The correlations between ZEB2 expression and survival in ovarian cancer were examined and presented with the hazard ratio, 95% confidence intervals, and log rank *p*-value computed.

### 2.3. PrognoScan Database Analysis

The PrognoScan database is widely used to evaluate biological relationships between gene expression and survival rates, such as disease-free survival, overall survival, and relapse-free survival [25]. It includes a large-scale collection of publicly available cancer microarray datasets with clinical information. We used this database to identify the correlation between ZEB2 mRNA expression and survival in ovarian cancer with a Cox *p*-value < 0.05.

### 2.4. Tumor Immune Estimation Resource (TIMER) Analysis

TIMER is used to analyze tumor-infiltrating immune cells in ovarian cancer [26]. TIMER assesses the tumor-infiltrating immune cells based on the statistical analysis of gene expression profiles [27]. We analyzed the correlation between ZEB2 and infiltrating immune cells, such as CD4+T cells, CD8+T cells, neutrophils, macrophages, dendritic cells, and B cells in different cancer types, including ovarian cancer.

### 2.5. STRING Analysis

The STRING protein network database can aid in the prediction of potential protein interactions. It is a precomputed global resource for the exploration and analysis of protein interactions. We used the STRING database to investigate whether ZEB2 has any protein-protein interactions.

### 2.6. Statistical Analysis

All statistical analyses were performed using the Statistical Package for the Social Sciences (SPSS), version 25.0, for Windows (IBM, Armonk, NY, USA). Survival results are presented with *p*-values from a log-rank test, and Kaplan–Meier survival curves were generated using PrognoScan online tools. The correlation of gene expression was evaluated in the TIMER database using Spearman’s correlation analysis. A two-tailed *p*-value of <0.05 was considered to indicate statistical significance.

## 3. Results

### 3.1. mRNA Levels of ZEB2 in Various Types of Cancer

**Figure 1:**
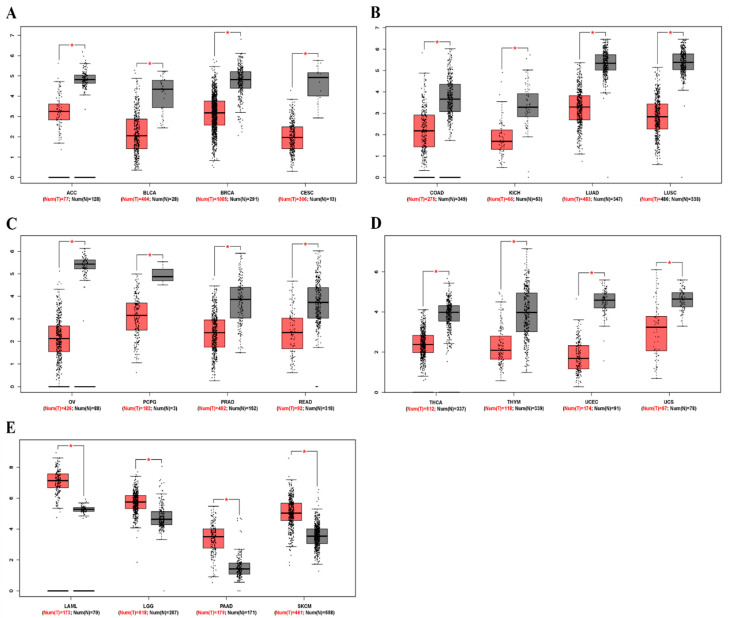
The mRNA levels of ZEB2 in different types of cancer. The expression levels of ZEB2 were analyzed using the GEPIA database. (**A**–**D**) Low expression of ZEB2 in various cancer tissues compared with normal tissue. (**E**) High expression of ZEB1 in various cancer tissues compared with normal tissue. Adrenocortical carcinoma, ACC; bladder urothelial carcinoma, BLCA; breast invasive carcinoma, BRCA; cervical squamous cell carcinoma, CESC; colon adenocarcinoma, COAD; kidney chromophobe, KICH; lung adenocarcinoma, LUAD; lung squamous cell carcinoma, LUSC; ovarian serous cystadenocarcinoma, OV; pheochromocytoma and paraganglioma, PCPG; prostate adenocarcinoma, PRAD; rectum adenocarcinoma, READ; thyroid carcinoma, THCA; thymoma, THYM; uterine corpus endometrial carcinoma, UCEC; uterine carcinosarcoma, UCS; acute myeloid leukemia, LAML; brain lower grade glioma, LGG; pancreatic adenocarcinoma, PAAD; skin cutaneous melanoma, SKCM (* *p* < 0.05).

To determine differences in ZEB2 mRNA expression between tumor and normal tissues, ZEB2 expression in normal samples and multiple cancer types, including ovarian cancer, was analyzed using the GEPIA database. The results revealed that ZEB2 expression was lower in adrenocortical carcinoma (ACC), bladder urothelial carcinoma (BLCA), breast invasive carcinoma (BRCA), cervical squamous cell carcinoma (CESC), colon adenocarcinoma (COAD), kidney chromophobe (KICH), lung adenocarcinoma (LUAD), lung squamous cell carcinoma (LUSC), ovarian serous cystadenocarcinoma (OV), pheochromocytoma and paraganglioma (PCPG), prostate adenocarcinoma (PRAD), rectum adenocarcinoma (READ), thyroid carcinoma (THCA), thymoma (THYM), uterine corpus endometrial carcinoma (UCEC), and uterine carcinosarcoma (UCS) than in normal tissue. However, ZEB2 expression was higher in acute myeloid leukemia (LAML), brain lower grade glioma (LGG), pancreatic adenocarcinoma (PAAD), and skin cutaneous melanoma (SKCM) than in normal tissue (Figure 1). These results suggest that the expression of ZEB2 was lower in OV compared to normal tissue. In addition, it was confirmed that the mRNA level of ZEB2 was different in various cancers.

### 3.2. The Prognostic Value of ZEB2 Expression in Various Types of Cancer

**Figure 2:**
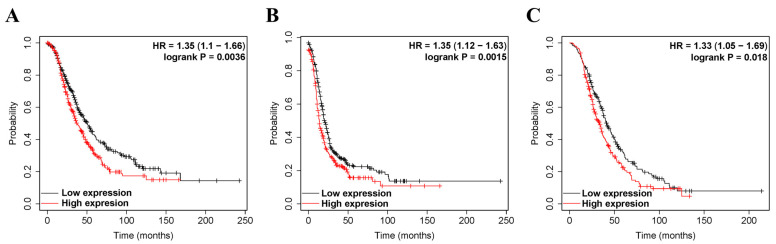
The prognostic significance of high expression of ZEB2 in cancers. The prognostic value of ZEB2 was analyzed using the Kaplan–Meier plotter. Survival curves OS (**A**), PFS (**B**), and PPS (**C**) of ZEB2 in OV. Overall survival, OS; progression-free survival, PFS; post-progression survival, PPS; ovarian serous cystadenocarcinoma, OV.

We investigated whether ZEB2 expression correlated with the prognosis of OV. Therefore, the effect of ZEB2 expression on survival rates was evaluated using the Kaplan–Meier plotter and PrognoScan databases. Survival rates, such as PFS, OS, and PPS of ZEB2 in OV, were analyzed. The findings revealed that high ZEB2 expression had significantly shorter survival times than those with low expression (Figure 2). High ZEB2 expression was associated with poorer prognosis in OV (PFS, HR = 1.35, *p* = 0.0015; OS, HR = 1.35, *p* = 0.0036; PPS, HR = 1.33, *p* = 0.0018; Figure 2A–C). These findings demonstrate the prognostic significance of ZEB2 in OV. Next, we investigated the relationship between ZEB2 and the clinicopathological characteristics of ovarian cancer using the Kaplan–Meier Plotter database, and the results are shown in Table 1. High ZEB2 expression correlated with poorer PFS in stages II (HR = 2.5, *p* = 0.043), II + III (HR = 1.34, *p* = 0.0056), II + III + IV (HR = 1.33, *p* = 0.0034), III (HR = 1.29, *p* = 0.017), III + IV (HR = 1.37, *p* = 0.0017), and IV (HR = 1.37, *p* = 0.0017). In addition, high ZEB2 expression correlated with poorer OS in stages II + III + IV (HR = 1.29, *p* = 0.025) and III + IV (HR = 1.26, *p* = 0.048). High ZEB2 expression correlated with poorer PPS in stages II + III (HR = 1.31, *p* = 0.041) and II + III + IV (HR = 1.29, *p* = 0.034). High ZEB2 expression correlated with poorer PFS, OS, and PPS in Grade II + III (PFS, HR = 1.33, *p* = 0.0061; OS, HR = 1.31, *p* = 0.0016; PPS, HR = 1.31, *p* = 0.033) and III (PFS, HR = 1.38, *p* = 0.012; OS, HR = 1.29, *p* = 0.044; PPS, HR = 1.37, *p* = 0.035). These findings revealed the prognostic significance of ZEB2 expression based on clinicopathological characteristics, especially in Grade II + III and Grade III OV. Moreover, the tumor suppressor gene p53 (TP53) mutation was associated with poorer OS in the wild type (HR = 2.83, *p* = 0.046). To further examine the prognostic potential of ZEB2 in different cancer types, we analyzed the PrognoScan database. Poor prognosis was identified in cancers of the blood, brain, breast, colorectal, and lung cancers, including ovarian cancer (Supplementary Table S1). Taken together, ZEB2 expression was associated with poorer prognosis in OV and other cancers.

### 3.3. Correlation between ZEB2 and Infiltrating Immune Cells in Various Types of Cancer

**Figure 3:**
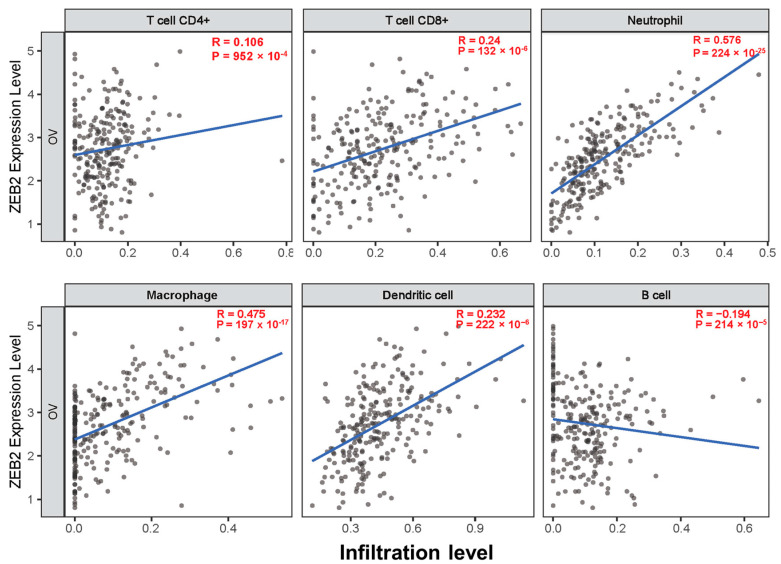
Correlation between ZEB2 expression and infiltrating immune cells in ovarian cancer. The correlation between ZEB2 and infiltrating immune cells (CD4+T cells, CD8+T cells, neutrophils, macrophages, dendritic cells, and B cells) was analyzed using the TIMER database.

The survival times of patients with several cancers are determined by the quantity and activity status of tumor-infiltrating lymphocytes [28,29]. We explored the correlation between ZEB2 and infiltrating immune cells in various cancers, including OV, using the TIMER database. We analyzed this correlation in various types of cancer, including OV. The results revealed that ZEB2 was significantly positively correlated with the infiltration levels of CD8+T cells (R = 0.24, *p* = 132 × 10^−6^), neutrophils (R = 0.576, *p* = 224 × 10^−25^), macrophages (R = 0.475, *p* = 197 × 10^−17^), and dendritic cells (R = 0.232, *p* = 222 × 10^−6^). However, ZEB2 was significantly negatively correlated with the infiltration levels of B cells (R = −0.194, *p* = 214 × 10^−5^) in OV (Figure 3). Moreover, ZEB2 correlates with the infiltration levels of CD4+T cells in 20 cancer types, CD8+T cells in 24 cancer types, B cells in 18 cancer types, neutrophils in 29 cancer types, macrophages in 26 cancer types, and dendritic cells in 28 cancer types (Supplementary Table S2). These findings suggest that ZEB2 expression correlates with the infiltration of immune cells in different cancer types, including OV.

### 3.4. Correlation between ZEB1 and ZEB2 Expression in Various Types of Cancer

To identify the correlation between ZEB1 and ZEB2 expression, we analyzed this correlation in 32 cancer types using the TIMER database (Table 2). Interestingly, these findings revealed that ZEB1 and ZEB2 were positively correlated in all cancer types.

### 3.5. Correlations with ZEB2-Related Proteins in Various Types of Cancer

**Figure 4:**
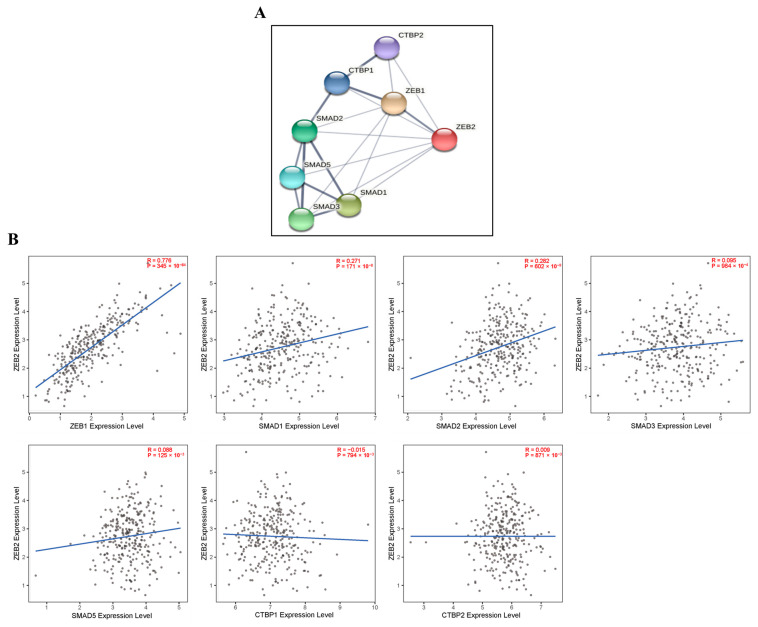
Correlations with ZEB2-related proteins in ovarian cancer. ZEB2 network database in OV was analyzed using the STRING online database (**A**). The correlation between ZEB2 and ZEB2-related proteins (ZEB1, SMAD1, SMAD2, SMAD3, SMAD5, CTBP1 and CTBP2) was analyzed using the TIMER database (**B**).

We investigated the correlations with ZEB2-related proteins using the TIMER and STRING databases. We analyzed this correlation in various types of cancer, including OV. These findings reveal that ZEB2 protein was positively correlated with ZEB1 (R = 0.776, *p* = 345 × 10^−64^), SMAD1 (R = 0.271, *p* = 171 × 10^−8^), and SMAD2 (R = 0.282, *p* = 602 × 10^−9^) in OV. However, ZEB2 did not significantly affect other SMADs proteins in OV. In addition, ZEB2 did not significantly affect CTBPs in the OV (Figure 4). Moreover, ZEB2 correlates with SMAD1 in 28 cancer types, SMAD2 in 27 cancer types, SMAD3 in 22 cancer types, SMAD5 in 28 cancer types, CTBP1 in 14 cancer types, and CTBP2 in 22 cancer types (Supplementary Table S3). These findings indicate that ZEB2 correlates with SMADs and CTBPs in various types of cancer. 

## 4. Discussion

OV, the most common gynecological malignancy, is characterized by a relatively high incidence and poor prognosis. In this study, the correlation with ZEB2 was confirmed by factor investigation to improve the prognosis of OV. ZEB2 is a transcription factor belonging to the human ZEB family. ZEB2 regulates gene expression by interacting with specific activators or repressors in various cancers. It is related to the development of cancer stem cell characteristics and treatment resistance, and is recognized as a reliable prognostic marker for cancer patient outcomes [30]. ZEB2 plays a significant role in EMT during tumor invasion and metastasis in a variety of human malignancies. EMT is a biological process characterized by the transformation of an epithelial cell phenotype to a mesenchymal phenotype, which is associated with increased cell motility and invasion. ZEB2 overexpression is known to have an aggressive correlation in a variety of cancers that may be involved in malignant transformation. Our results show that ZEB2 expression was lower in OV and many cancers compared to normal tissue. However, ZEB2 is relatively highly expressed in tumors such as leukemia, brain glioma, pancreatic adenocarcinoma, and melanoma. Previous studies suggest that ZEB2 is associated with a bad prognosis in numerous kinds of cancer [18,19,31]. Nevertheless, the prognostic significance of ZEB2 in OV remains unclear. Thus, we performed a comprehensive meta-analysis to evaluate the prognostic value of ZEB2 in different types of cancer, including OV. Our results show that high ZEB2 expression correlates with poorer PFS, OS, and PPS in OV. Moreover, it correlates with worse PFS, OS, and PPS in grades II + III and III. This result is in agreement with previous studies in OV patients, suggesting its metastatic potential [18,19]. Although its expression was decreased in OV compared to normal tissue, higher EZH2 expression induced peritoneal metastasis, such as cancer stem cells. The tumor-suppressing gene TP53 is involved in cell cycle control and apoptosis after DNA damage. However, when mutations occur, DNA-damaged cells can escape apoptosis and turn into cancer cells [32]. We showed that TP53 mutations were associated with unfavorable OS in the wild type. These findings suggest that ZEB2 is a potential prognostic marker for OV.

Cancer is caused by a variety of causes over a long period of time and is originally caused by cancer cells avoiding the immune system with the advantage of being one’s own cells. Moreover, these cancer cells are known to affect the onset, growth, and metastasis of tumors by interacting with immune cells, such as T and B lymphocytes, macrophages, neutrophils, and dendritic cells in the body [33]. Cancer cells, by engaging in a dynamic crosstalk with immune cells, exhibit EMT plasticity to adapt to the changing microenvironment they encounter in the primary tumor, during metastasis, and at distant sites. Tumorigenicity and invasiveness are important acquired characteristics for the development and progression of cancer, and could be regulated by transcription factors associated with EMT, such as ZEB1, ZEB2, SNAI1, SLUG, and STAT3 [34]. Elevated ZEB2 is known to correlate with the acquisition and function of CSCs. Moreover, ZEB2 plays an essential role in NK cell maturation, CD8+T cell differentiation, and dendritic cell development [35,36,37]. To the best of our knowledge, our study is the first to explore the correlation between ZEB2 and the immune infiltrate in OV. Our findings reveal that ZEB2 is positively correlated with the infiltration levels of CD8+T cells, neutrophils, macrophages, and dendritic cells in OV. However, ZEB2 is negatively correlated with B cell infiltration levels. These findings suggest that ZEB2 may be a potential diagnostic and therapeutic target in patients with OV. Further research is needed to confirm the correlation between ZEB2 expression and infiltrating immune cells.

ZEB1 and ZEB2 have many similarities in transcriptional regulation; they have different expression patterns and molecular and biological roles, such as cell differentiation and disease progression regulation [38,39]. Both ZEB1 and ZEB2 induce EMT and enhance cancer progression. They transform into mesenchymal cells during the EMT process; epithelial cells lose their adhesiveness and become migratory and invasive [40,41]. TGF-β is a crucial cytokine that promotes EMT [42]. TGF-β promotes their expression in some types of normal and malignant cells, as well as certain other EMT-related transcription factors such as Snail and Slug [43,44]. TGF-β reduces E-cadherin expression in mammary epithelial cells via inducing ZEB1 and ZEB2. In recent years, ZEB1 and ZEB2 have been found in a variety of malignancies [45,46]. However, the correlation between ZEB1 and ZEB2 in cancer is yet to be elucidated. Herein, we identified a correlation between ZEB1 and ZEB2 in various cancers. Interestingly, our findings reveal that ZEB2 has a positive correlation in all cancers. ZEB1 and ZEB2 may have multiple functions that will be elucidated by analyses of specific cancer types in the future. The detailed mechanisms of ZEB1 and ZEB2 in cancer should be elucidated.

According to published studies, EMT is an essential mechanism for tumor progression, intrusion, and metastasis, and ZEB2 has been reported as a key transcription factor for EMT [41,47]. ZEB2 has been identified as a protein that interacts with receptor-active SMAD in the pathway related to signals from other members of the TGF-β superfamily [48]. In addition, the TGF-β pathway has been found in breast cancer, non-small cell lung cancer, and other tumors that promote EMT progression [49]. TGF-β stimulates the expression of other EMT-related transcription factors. TGF-β binds to type I and type II receptors, and transduces signals via SMAD and non-SMAD signaling pathways. The TGF-β type I receptor is activated by ligand stimulation and phosphorylates the receptor-regulated SMADs, SMAD2, and SMAD3, which form trimeric complexes with the common partner SMAD and SMAD4. Activation of TGF-β receptors causes the phosphorylation and nuclear translocation of SMAD proteins, which then participate in the regulation of target gene expression. ZEB2 can also mediate transcriptional repression via cooperation with activated SMADs, or through recruitment of the corepressor CTBP, as well as histone deacetylase complexes [50]. Our results show that ZEB2 was positively correlated with SMAD1 and SMAD2 proteins in OV.

## 5. Conclusions

In conclusion, our results demonstrate a lower expression of ZEB2 in OV; however, patients with high ZEB2 expression may induce poor prognosis. To understand its clinical significance, the criteria of high ZEB2 expression should be investigated in OV, since the majority of OV did not have ZEB2 expression. Moreover, we suggest that increased ZEB2 expression is correlated with infiltrating immune cells and SMAD1 and SMAD2 in OV. Taken together, our findings suggest that ZEB2 could be a potential prognostic biomarker and may provide novel insights into tumor immunology. However, depending on intratumor heterogeneity, the expression level and prognosis of numerous genes may differ even within tumors. Therefore, transcriptome analysis of single cancer cells in tumor heterogeneity will be required to demonstrate the possibility as a potential prognostic biomarker of ZEB2 in various cancers including OV. In addition, the detailed mechanisms by which ZEB2 contributes to the correlation in cancer should be elucidated further. 

## Figures and Tables

**Table 1:** Association between ZEB2 and clinicopathological characteristics in ovarian cancer. The clinicopathological characteristics of ZEB2 were analyzed using the Kaplan–Meier plotter.

Clinicopathological Characteristics	Progression-Free Survival (*n* = 614)			Overall Survival (*n* = 1144)			Post-progression Survival (*n* = 138)		
	*n*	Hazard Ratio	*p*-Value	*n*	Hazard Ratio	*p*-Value	*n*	Hazard Ratio	*p*-Value
STAGE									
I	74	1.3 (0.36–4.7)	0.68	51	1.57 (0.39–6.32)	0.52	7	-	-
I+ II	115	2.13 (0.94–4.8)	0.064	83	1.2 (0.43–3.4)	0.73	20	1.85 (0.52–6.7)	0.33
II	14	2.5 (1.0–6.4)	0.043	32	2.7 (0.52–14)	0.22	13	5.26 (0.59–48)	0.093
II + III	465	1.34 (1.09–1.64)	**0.0056**	458	1.21 (0.95–1.54)	0.13	325	1.31 (1.01–1.7)	**0.041**
II + III + IV	535	1.33 (1.1–1.61)	**0.0034**	519	1.29 (1.03–1.61)	**0.025**	374	1.29 (1.02–1.64)	**0.034**
III	424	1.29 (1.05–1.6)	**0.017**	426	1.26 (0.99–1.62)	0.063	312	1.25 (0.96–1.62)	0.097
III + IV	494	1.37 (1.12–1.66)	**0.0017**	487	1.26 (1.0–1.58)	**0.048**	361	1.22 (0.96–1.56)	0.099
IV	70	1.65 (0.99–2.75)	**0.05**	61	1.13 (0.63–2.01)	0.69	49	1.1 (0.59–2.1)	0.76
GRADE									
I	54	4.16 (0.86–20)	0.054	41	1.12 (0.39–3.22)	0.84	9	-	-
I+ II	189	1.38 (0.97–1.96)	0.074	203	1.17 (0.78–1.75)	0.44	118	1.12 (0.72–1.74)	0.62
II	161	1.27 (0.88–1.83)	0.19	162	1.18 (0.76–1.83)	0.46	109	1.09 (0.68–1.74)	0.71
II + III	476	1.33 (1.08–1.64)	**0.0061**	554	1.31 (1.05–1.62)	**0.016**	349	1.31 (1.02–1.68)	**0.033**
III	315	1.38 (1.07–1.77)	**0.012**	392	1.29 (1.01–1.66)	**0.044**	240	1.37 (1.02–1.84)	**0.035**
IV	18	-	-	18	1.06 (0.39–2.9)	0.91	18	-	-
TP53 mutation									
Mutated	124	1.26 (0.87–1.84)	0.22	124	1.18 (0.81–1.73)	0.38	116	1.02 (0.7–1.5)	0.92
Wild type	19	2.2 (0.77–6.33)	0.13	19	2.83 (0.98–8.16)	**0.046**	17	2.02 (0.7–5.8)	0.18

Bold values indicate *p* < 0.05.

**Table 2:** Correlation between ZEB1 and ZEB2 expression in various types of cancer.

Cancer Type	R	*p*
Adrenocortical carcinoma	0.30	**0.007**
Bladder urothelial carcinoma	0.82	**0.000**
Breast invasive carcinoma	0.80	**0.000**
Cervical squamous cell carcinoma andendocervical adenocarcinoma	0.71	**0.000**
Cholangiocarcinoma	0.70	**0.000**
Colon adenocarcinoma	0.85	**0.000**
Lymphoid neoplasm diffuse large B-cellLymphoma	0.79	**0.000**
Esophageal carcinoma	0.87	**0.000**
Glioblastoma multiforme	0.49	**0.000**
Head and neck squamous cell carcinoma	0.87	**0.000**
Kidney chromophobe	0.71	**0.000**
Kidney renal clear cell carcinoma	0.75	**0.000**
Kidney renal papillary cell carcinoma	0.47	**0.000**
Brain lower grade glioma	0.54	**0.000**
Liver hepatocellular carcinoma	0.57	**0.000**
Lung adenocarcinoma	0.78	**0.000**
Lung squamous cell carcinoma	0.76	**0.000**
Mesothelioma	0.51	**0.000**
Ovarian serous cystadenocarcinoma	0.78	**0.000**
Pancreatic adenocarcinoma	0.88	**0.000**
Pheochromocytoma and paraganglioma	0.12	**0.097**
Prostate adenocarcinoma	0.86	**0.000**
Rectum adenocarcinoma	0.89	**0.000**
Sarcoma	0.14	**0.029**
Skin cutaneous melanoma	0.20	**0.000**
Stomach adenocarcinoma	0.82	**0.000**
Testicular germ cell tumors	0.76	**0.000**
Thyroid carcinoma	0.69	**0.000**
Thymoma	0.22	**0.017**
Uterine corpus endometrial carcinoma	0.71	**0.000**
Uterine carcinosarcoma	0.52	**0.000**
Uveal melanoma	0.61	**0.000**

Bold values indicate *p* < 0.05.

## Data Availability

Not applicable.

## Supplementary Materials

The following supporting information can be downloaded at: https://www.mdpi.com/article/10.3390/cimb44030079/s1, Table S1: Survival analysis of ZEB2 in various cancers using prognoscan database. Table S2: Correlation between ZEB2 and infiltrating immune cells in different types of cancer using TIMER database. Table S3: Correlations with ZEB2-related proteins in various types of cancer using STRING network database.
