# Supplementary material for: Zinc Finger E-Box Binding Homeobox 2 as a Prognostic Biomarker in Various Cancers and Its Correlation with Infiltrating Immune Cells in Ovarian Cancer
Source: Curr Issues Mol Biol. 2022 Mar 1;44(3):1203–14. doi: 10.3390/cimb44030079 (PMC8947672; doi:10.3390/cimb44030079)
Supplement: Supplementary file 1 [file cimb-44-00079-s001.zip › cimb-1598248-supplementary.pdf]

Supplementary Materials:

Supplementary Table S1. Survival analysis of ZEB2 in various cancers using prognoscan database.

DATASET	CANCER TYPE	ENDPOINT	*N*	ln(HR-high / HR-low)	*p*-VALUE	ln(HR)	HR [95% CI-low CI-up]
GSE16131-GPL97	Blood cancer	Overall Survival	180	0.806407	**0.0470009**	0.446249	1.56 [1.01 - 2.43]
GSE4412-GPL96	Brain cancer	Overall Survival	74	-1.2724	**0.0404328**	-0.634978	0.53 [0.29 - 0.97]
GSE16581	Brain cancer	Overall Survival	67	1.80158	**0.0376143**	2.76856	15.94 [1.17 - 216.72]
GSE19615	Breast cancer	Distant Metastasis Free Surviva	115	-1.77523	**0.0293108**	-1.0131	0.36 [0.15 - 0.90]
GSE12276	Breast cancer	Relapse Free Survival	204	-0.563349	**0.0328363**	-0.272153	0.76 [0.59 - 0.98]
GSE1456-GPL96	Breast cancer	Disease Specific Survival	159	-1.30938	**0.0458268**	-0.633631	0.53 [0.28 - 0.99]
GSE3494-GPL96	Breast cancer	Disease Specific Survival	236	-1.07136	**0.0377182**	-0.533531	0.59 [0.35 - 0.97]
GSE4922-GPL96	Breast cancer	Disease Free Survival	249	-1.00845	**0.0201433**	-0.464822	0.63 [0.42 - 0.93]
GSE4922-GPL97	Breast cancer	Disease Free Survival	249	-0.742581	**0.0238131**	-0.516104	0.60 [0.38 - 0.93]
GSE17536	Colorectal cancer	Disease Free Survival	145	1.24824	**0.038437**	0.898199	2.46 [1.05 - 5.75]
GSE17536	Colorectal cancer	Disease Free Survival	145	2.08083	**0.0032525**	0.60329	1.83 [1.22 - 2.73]
GSE17536	Colorectal cancer	Disease Specific Survival	177	0.840664	**0.00405702**	0.463378	1.59 [1.16 - 2.18]
GSE17536	Colorectal cancer	Disease Specific Survival	177	-1.26034	**0.0373419**	-2.16037	0.12 [0.02 - 0.88]
GSE17536	Colorectal cancer	Disease Specific Survival	177	0.953897	**0.0241088**	0.785333	2.19 [1.11 - 4.34]
GSE17536	Colorectal cancer	Overall Survival	177	-1.13161	**0.0179672**	-2.11959	0.12 [0.02 - 0.69]
GSE17536	Colorectal cancer	Overall Survival	177	0.764409	**0.0177957**	0.325741	1.39 [1.06 - 1.81]
GSE14333	Colorectal cancer	Disease Free Survival	226	1.02136	**0.0261089**	0.251215	1.29 [1.03 - 1.60]
GSE14333	Colorectal cancer	Disease Free Survival	226	0.969175	**0.0195668**	0.310015	1.36 [1.05 - 1.77]
GSE14333	Colorectal cancer	Disease Free Survival	226	1.1729	**0.00513886**	0.506459	1.66 [1.16 - 2.37]
GSE31210	Lung cancer	Relapse Free Survival	204	-0.845839	**0.00169391**	-0.956522	0.38 [0.21 - 0.70]
GSE9891	Ovarian cancer	Overall Survival	278	-0.809959	**0.0447499**	-1.12764	0.32 [0.11 - 0.97]

Bold values indicate $P < 0.05$

Supplementary Table S2. Correlation between ZEB2 and infiltrating immune cells in different types of cancer using TIMER database.

Cancer type	T-cell CD4+		T-cell CD8+		B Cell		Nutrophil		Macrophage		Dendritic cell	
	R	P	R	P	R	P	R	P	R	P	R	P
Adrenocortical carcinoma	-0.06	0.616	0.18	0.132	0.21	0.068	0.15	0.208	0.27	**0.020**	0.07	0.580
Bladder Urothelial Carcinoma	0.17	**0.001**	0.25	**0.000**	-0.18	**0.000**	0.37	**0.000**	0.39	**0.000**	0.30	**0.000**
Breast invasive carcinoma	0.04	0.268	0.34	**0.000**	-0.17	**0.000**	0.58	**0.000**	0.50	**0.000**	0.42	**0.000**
Cervical squamous cell carcinoma and endocervical adenocarcinoma	0.21	**0.000**	0.29	**0.000**	0.09	0.134	0.36	**0.000**	0.20	**0.001**	0.44	**0.000**
Cholangiocarcinoma	0.15	0.396	0.48	**0.003**	0.47	**0.005**	0.21	0.232	0.41	**0.014**	0.60	**0.000**
Colon adenocarcinoma	0.43	**0.000**	0.32	**0.000**	-0.19	**0.002**	0.70	**0.000**	0.61	**0.000**	0.80	**0.000**
Lymphoid Neoplasm Diffuse Large B-cell Lymphoma	-0.12	0.472	0.51	**0.001**	-0.03	0.867	0.82	**0.000**	-0.16	0.329	0.32	**0.038**
Esophageal carcinoma	0.23	0.002	0.36	**0.000**	0.15	**0.048**	0.38	**0.000**	0.65	**0.000**	0.53	**0.000**
Glioblastoma multiforme	0.06	0.484	-0.16	0.069	0.30	**0.000**	0.03	0.689	0.10	0.260	0.23	**0.006**
Head and Neck squamous cell carcinoma	0.38	**0.000**	-0.25	**0.000**	-0.34	**0.000**	0.49	**0.000**	0.57	**0.000**	0.25	**0.000**
Kidney Chromophobe	0.06	0.657	0.41	**0.001**	0.48	**0.000**	0.36	**0.003**	0.58	**0.000**	0.64	**0.000**
Kidney renal clear cell carcinoma	0.42	**0.000**	0.09	**0.044**	-0.26	**0.000**	0.62	**0.000**	0.50	**0.000**	0.25	**0.000**
Kidney renal papillary cell carcinoma	0.15	**0.019**	0.30	**0.000**	0.30	**0.000**	0.44	**0.000**	0.41	**0.000**	0.44	**0.000**
Brain Lower Grade Glioma	-0.10	**0.031**	0.11	0.014	0.22	**0.000**	0.10	**0.036**	-0.04	0.422	-0.09	**0.038**
Liver hepatocellular carcinoma	0.30	**0.000**	0.43	**0.000**	0.30	**0.000**	0.38	**0.000**	0.55	**0.000**	0.61	**0.000**
Lung adenocarcinoma	0.18	**0.000**	0.39	**0.000**	-0.04	0.403	0.62	**0.000**	0.53	**0.000**	0.45	**0.000**
Lung squamous cell carcinoma	0.20	**0.000**	0.37	**0.000**	0.05	0.326	0.40	**0.000**	0.26	**0.000**	0.58	**0.000**
Mesothelioma	0.04	0.714	0.31	**0.003**	0.10	0.355	0.30	**0.006**	0.57	**0.000**	0.50	**0.000**
Ovarian serous cystadenocarcinoma	0.11	0.095	0.24	**0.000**	-0.19	**0.002**	0.58	**0.000**	0.48	**0.000**	0.23	**0.000**
Pancreatic adenocarcinoma	0.08	0.286	0.72	**0.000**	0.06	0.447	0.72	**0.000**	0.68	**0.000**	0.78	**0.000**
Pheochromocytoma and Paraganglioma	0.26	**0.001**	0.25	**0.001**	0.01	0.929	0.34	**0.000**	0.29	**0.000**	0.48	**0.000**
Prostate adenocarcinoma	0.52	**0.000**	0.18	**0.000**	-0.02	0.629	0.61	**0.000**	0.33	**0.000**	0.67	**0.000**
Rectum adenocarcinoma	0.46	**0.000**	0.36	**0.000**	0.12	0.265	0.56	**0.000**	0.57	**0.000**	0.80	**0.000**
Sarcoma	0.24	**0.000**	0.03	0.690	-0.05	0.432	0.41	**0.000**	0.28	**0.000**	0.13	**0.042**
Skin Cutaneous Melanoma	0.15	**0.001**	0.09	0.056	-0.04	0.452	0.49	**0.000**	0.09	0.051	0.04	0.449
Stomach adenocarcinoma	0.40	**0.000**	0.62	**0.000**	0.13	**0.012**	0.60	**0.000**	0.73	**0.000**	0.62	**0.000**
Testicular Germ Cell Tumors	0.45	**0.000**	-0.08	0.341	-0.38	**0.000**	0.47	**0.000**	0.17	**0.037**	0.38	**0.000**
Thyroid carcinoma	0.48	**0.000**	-0.26	**0.000**	-0.18	**0.000**	0.39	**0.000**	0.27	**0.000**	0.24	**0.000**
Thymoma	-0.23	**0.012**	-0.46	**0.000**	-0.13	0.161	0.25	**0.007**	0.27	**0.004**	-0.25	**0.008**
Uterine Corpus Endometrial Carcinoma	0.37	**0.000**	0.20	0.063	0.34	**0.001**	0.48	**0.000**	0.51	**0.000**	0.27	**0.012**
Uterine Carcinosarcoma	-0.25	0.066	0.39	**0.004**	-0.01	0.961	-0.34	**0.012**	0.14	0.327	0.15	0.288
Uveal Melanoma	-0.10	0.379	0.11	0.335	-0.29	**0.010**	0.28	**0.015**	0.08	0.470	-0.02	0.891

Bold values indicate P < 0.05

Supplementary Table S3. Correlations with ZEB2-related proteins in various types of cancer using STRING network database.

Cancer type	SMAD1		SMAD2		SMAD3		SMAD5		CTBP1		CTBP2	
	R	P	R	P	R	P	R	P	R	P	R	P
Adrenocortical carcinoma	0.51	**0.000**	0.46	**0.000**	0.41	**0.000**	0.44	**0.000**	0.18	0.120	0.38	**0.001**
Bladder Urothelial Carcinoma	0.31	**0.000**	0.04	0.378	-0.23	**0.000**	-0.03	0.558	-0.10	0.053	0.13	**0.006**
Breast invasive carcinoma	0.41	**0.000**	0.31	**0.000**	0.28	**0.000**	0.35	**0.000**	-0.09	**0.003**	0.15	**0.000**
Cervical squamous cell carcinoma and endocervical adenocarcinoma	0.17	**0.003**	0.18	**0.002**	0.02	0.777	0.20	**0.000**	0.07	0.233	-0.12	**0.030**
Cholangiocarcinoma	0.56	**0.000**	0.20	0.247	-0.08	0.642	0.05	0.786	-0.03	0.844	-0.04	0.836
Colon adenocarcinoma	0.51	**0.000**	0.41	**0.000**	0.32	**0.000**	0.37	**0.000**	-0.04	0.434	0.16	**0.001**
Lymphoid Neoplasm Diffuse Large B-cell Lymphoma	0.51	**0.000**	0.75	**0.000**	0.63	**0.000**	0.70	**0.000**	0.20	0.175	0.69	**0.000**
Esophageal carcinoma	0.19	**0.010**	0.11	0.142	0.02	0.781	0.26	**0.000**	0.02	0.822	-0.12	0.100
Glioblastoma multiforme	0.11	0.168	0.34	**0.000**	0.17	**0.035**	0.39	**0.000**	0.43	**0.000**	0.51	**0.000**
Head and Neck squamous cell carcinoma	0.38	**0.000**	0.37	**0.000**	0.10	**0.026**	0.42	**0.000**	0.28	**0.000**	0.13	**0.002**
Kidney Chromophobe	0.14	0.245	0.29	**0.017**	-0.02	0.872	0.39	**0.001**	0.05	0.705	0.39	**0.001**
Kidney renal clear cell carcinoma	0.57	**0.000**	0.59	**0.000**	0.35	**0.000**	0.61	**0.000**	0.06	0.164	0.49	**0.000**
Kidney renal papillary cell carcinoma	0.51	**0.000**	0.41	**0.000**	0.25	**0.000**	0.52	**0.000**	0.26	**0.000**	0.46	**0.000**
Brain Lower Grade Glioma	0.28	**0.000**	0.48	**0.000**	0.05	0.246	0.53	**0.000**	-0.07	0.116	0.44	**0.000**
Liver hepatocellular carcinoma	0.46	**0.000**	0.44	**0.000**	0.26	**0.000**	0.46	**0.000**	0.18	**0.001**	0.70	**0.000**
Lung adenocarcinoma	0.31	**0.000**	0.19	**0.000**	0.24	**0.000**	0.29	**0.000**	-0.14	**0.002**	0.00	0.949
Lung squamous cell carcinoma	0.19	**0.000**	0.11	**0.015**	-0.06	0.189	0.12	**0.008**	0.07	0.113	0.01	0.809
Mesothelioma	0.47	**0.000**	0.40	**0.000**	0.22	**0.039**	0.35	**0.001**	-0.10	0.374	0.41	**0.000**
Ovarian serous cystadenocarcinoma	0.27	**0.000**	0.28	**0.000**	0.10	0.098	0.09	0.125	-0.02	0.794	0.01	0.871
Pancreatic adenocarcinoma	0.64	**0.000**	0.65	**0.000**	0.03	0.738	0.53	**0.000**	-0.11	0.136	0.04	0.553
Pheochromocytoma and Paraganglioma	0.40	**0.000**	0.10	0.186	-0.03	0.701	0.12	0.096	-0.27	**0.000**	0.35	**0.000**
Prostate adenocarcinoma	0.58	**0.000**	0.44	**0.000**	0.72	**0.000**	0.47	**0.000**	-0.11	**0.011**	0.20	**0.000**
Rectum adenocarcinoma	0.48	**0.000**	0.44	**0.000**	0.42	**0.000**	0.47	**0.000**	-0.16	**0.037**	0.11	0.157
Sarcoma	0.08	0.193	0.15	**0.017**	0.31	**0.000**	0.21	**0.001**	-0.14	**0.021**	0.11	0.077
Skin Cutaneous Melanoma	0.21	**0.000**	0.48	**0.000**	0.29	**0.000**	0.56	**0.000**	0.19	**0.000**	0.47	**0.000**
Stomach adenocarcinoma	0.36	**0.000**	0.28	**0.000**	0.11	**0.026**	0.40	**0.000**	-0.04	0.410	-0.12	**0.013**
Testicular Germ Cell Tumors	0.59	**0.000**	0.35	**0.000**	0.53	**0.000**	0.35	**0.000**	0.53	**0.000**	-0.16	**0.050**
Thyroid carcinoma	0.38	**0.000**	0.10	**0.025**	0.15	**0.001**	0.31	**0.000**	0.03	0.546	0.08	0.075
Thymoma	0.55	**0.000**	0.48	**0.000**	0.61	**0.000**	0.45	**0.000**	-0.43	**0.000**	0.32	**0.000**
Uterine Corpus Endometrial Carcinoma	0.38	**0.000**	0.17	**0.000**	0.21	**0.000**	0.29	**0.000**	-0.05	0.244	0.09	**0.040**
Uterine Carcinosarcoma	0.19	0.159	0.04	0.788	0.24	0.078	0.28	**0.038**	-0.08	0.531	0.05	0.717
Uveal Melanoma	0.73	**0.000**	0.73	**0.000**	0.50	**0.000**	0.72	**0.000**	0.51	**0.000**	0.77	**0.000**

Bold values indicate $P < 0.05$
